# Notes and News

**Published:** 1890-12-06

**Authors:** 


### Notes and News.

Collected and Communicated.

A very "swell" bazaar under Royal patronage will be held at 30, Wimpole Street, on December 10th, in aid of the French Hospital. All the stall-holders will be in fancy costume, and admission will be solely by ticket. Rumour whispers that some of our prettiest actresses will take an active part at this bazaar.

Torquay can boast another home for convalescents, and truly it is the very town where such homes should abound. "Ockenden," in Warren Road, formerly the residence of the donor, Miss Erle, is situate on the spur of Waldon Hill, and is at a high altitude. The home was opened by the Hon. Lady Northcote on November 1st, and is primarily intended for those who have been patients at the Devon and Exeter Hospital, or other Exeter charities, but the committee retain power to admit anyone likely to be benefited. There are in all fifteen rooms, and accommodation is afforded for twelve patients, who have the convenience of a conservatory, secluded garden, and well-protected verandah, and separate bed-rooms. Miss Erle has not only given the house, but has also promised to defray the rates and taxes, besides subscribing towards the maintenance fund.

Worcester is a town of progress, they have erected there two Victoria Kitchens, one of which was opened last week by Dr. Strange. In his opening address Dr. Strange said it was four years ago since the movement was started in Worcester. It was promoted to supply good cooked food, such as soup and pudding, to the poor people in the densely-populated parts of the city, where through smallness of houses, or ignorance in cooking, the people could not give their children good food. When food was prepared in large quantities it could be obtained more cheaply than it could be made at home. They now had six cooking kitchens, including one at St. John's, and five branches, all of them situated in poor and crowded neighbourhoods where the poor resided, and as near to the poor as they could get them. Another movement which flourishes in Worcester is the St. John Ambulance Association, and Lady Lechmere has presented many policemen with badges and medals and certificates awarded by this Association.

At the meeting of the Metropolitan Asylums Board, held in Spring Gardens on Saturday, Sir E. Galsworthy presiding, the fever returns showed that there are now 2,315 patients in the hospitals of the managers, as against 2,435 at the end the preceding fortnight; of this number 1,952 are suffering from scarlet fever. The General Purposes Committee recommended that provision should be made for a fever hospital for the relief of the Eastern Hospital; that it should contain accommodation for about four hundred patients; and that the General Purposes Committee be empowered to ascertain what eligible sites were available for the purpose. After a short discussion the resolution was adopted.

At the annual meeting in connection with the Manchester and Salford Hospital Sunday and Saturday Fund, it was reported that the amount paid to the Hospital Sunday account during the past year had been £4,617, against £4,636 last year; while the amount contributed to the Hospital Saturday Fund had been £3,657, against £3,536 a year ago. The amount raised for the medical charities by this organization during the past 21 years has been £154,770 14s. 10d. The Mayor of Manchester, who was in the chair, said he deplored the fact that surrounding towns did not subscribe to the funds to an extent commensurate with the benefits they received from the local charities.

A remarkable story of the restoration of an apparently drowned man was told to the Essex coroner last week. Three men were out in a boat—there was no dog there—which capsized; two were drowned, one was towed ashore apparently drowned, and a medical man pronounced life extinct. However, two men set to work to rub the body and used artificial respiration, and after two hours animation returned. The old motto of *nil desperandum* needs more frequent application in cases of supposed death; once or twice lately animation has been restored to those who have apparently succumbed to chloroform, after intervals of three, four, and in one case six hours. It is terrible to contemplate the number of persons who must have been allowed to pass into the Silent Land because hope was too quickly abandoned, and artificial respiration arrested after too short a time.

The troubles at Bristol General Hospital are settled by a compromise, and, as usual, no one is satisfied. Still there is peace now, and as the Committee had evidently done their best to please all parties it is to be hoped that all will try and cultivate friendly feelings and the spirit of content. The Committee will remain at its present number, but some of the junior members of the medical staff have courteously offered to retire to make room on the Committee for three members of the consulting staff. With regard to the qualifications of Scotch and Irish medical candidates the Committee could not determine this question, and it was most important to them that the disagreement should be got rid of, they had avoided the question altogether—passed it by. By this contrivance they opened all posts in the hospital to any candidates, whatever might be their qualifications, whatever their diplomas, who were legally, under the laws of the country, qualified to practise as physicians and surgeons. Dr. Stewart, at the last meeting, said the Royal College of Physicians and Surgeons of Ireland, which furnished a very large proportion of the medical men in practice in what they called the United Kingdom—certainly a United Kingdom in medical matters—had commissioned him to say while they accepted the concession that had been made in allowing alumni of the two colleges to compete for hospital appointments, they regretted it had been made in so ungenerous a way.

THE following vacancies are announced this week, the amount of the salary in each case being given after the name of the institution :—

**Resident Medical Officers.**

Chelsea Hospital for Sick Children, House Physician—£50, board, &c.
Miller Hospital, Greenwich, Senior Medical Officer—£60, board, &c.
Miller Hospital, Greenwich, Junior Medical Officer—£30, board, &c.
Denbigh Infirmary, House Surgeon—£85, board, &c.
East Riding Asylum, Assistant Medical Officer—£100, board, &c.
Royal Berks Hospital, Assistant House Surgeon—£40, board, &c.
Stafford County Asylum, Junior Assistant Medical Officer—£100, board, &c.
Birmingham Children's Hospital, Assistant Medical Officer—£40, board, &c.
Matlock Hydropathic, Junior Physician—£100, board, &c.

**Non-Resident Medical Officers.**

Western Dispensary, Westminster, Medical Officer.
Shadwell Hospital for Children, Secretary—£250.

---

WE record with deep regret the death of John Ring, jun., M.R.C.S., of Kilburn, on the 28th ult. Mr. Ring was student and house physician at Middlesex Hospital, and lately joined his father in his practice in North London. During the influenza epidemic Mr. Ring worked very hard, though himself a victim of the prevailing sickness. He never fully recovered the effects of the influenza, and during the summer symptoms of phthisis set in. Mr. Ring was a great favourite at the Middlesex, and his untimely death is deeply deplored.

---

THE Quarterly Court of the Brompton Consumption Hospital was held on Nov. 27th. The report of the Committee of Management regretted their inability to announce, as usual, the continued occupation of the 321 beds in the two buildings. The south wing having been professionally examined, some imperfections were discovered in some of the external drains. In order that no precaution should be neglected, the committee had placed the matter in the hands of Mr. Rogers Field, C.E., whose plans would be carried out with all possible despatch. The valuable weekly entertainments to the inmates had been resumed, the 24th annual season having commenced on the 4th inst. The resignation of Mr. H. H. Taylor, F.R.C.S., the resident medical officer, was much regretted; Dr. J. E. Kershaw had been elected as his successor, and Dr. J. S. Allden had been appointed assistant resident medical officer. Several legacies were acknowledged.

---

THE annual meeting of the Dudley Guest Hospital has been held. The report stated that the wave of better trade that had passed over the country had not been unmarked in the annals of the hospital, the year just ended showing an increase in the income of £336 2s. 1d. over the previous year. The amount of the workmen's collections had shown a marked increase, the total being £669 10s. 10d. The donations also showed a considerable increase owing to the generous gift of £200 by the Earl of Dudley towards furnishing the new wards. There was a decrease of £35 0s. 8d. in the Hospital Sunday collections. The new infectious wards, which were opened by the Earl of Dudley on February 8th, had already proved of great convenience in isolating infectious cases. During the year two nurses had been added to the nursing staff to undertake nursing cases in the town and district. The report of the medical staff stated that during the year 623 patients had been under treatment in the wards, and the large percentage of 69·02 had been discharged cured. The percentage of deaths for the same period was only 7·70. One hundred and thirty-three cases had been treated in the casualty-room.

DESPITE the telegrams from Pesth to the contrary, it appears that it is not the influenza, but typhoid fever which has broken out in Fuenzkirchen, the origin of the epidemic being foul drinking water. More than 2,000 persons are ill in the suburbs, but no one has been attacked whose water supply was from other wells.

---

DR. GEORGE ANDRÉ has been lecturing to medical men and students at Manchester on Hypnotism, ending with experiments, to which the students willingly lent themselves. Dr. André holds that hypnotism can be made of the greatest use both in surgery and medicine. At the close of his address and demonstration he was heartily thanked and applauded by the audience.

---

THERE was a nice case at the West Ham Police Court last week. A woman was summoned for allowing her children to be abroad while suffering from scarlet fever. It came out incidentally that the woman had actually brought the children to the court with her, having, as she simply explained, "nowhere to leave them and no one to take care of them." This is how infectious diseases are spread.

---

THE quarterly and annual court of governors of the London Hospital was held on Wednesday, and was very largely attended. The burning question of the efficiency of the nursing staff of course usurped the chief place in the proceedings. The report of the sub-committee to enquire into the recent allegations was read, and was eminently satisfactory. The members of the sub-committee had gone to ten large London hospitals and to the Edinburgh Infirmary, and compared their nursing system with these others; the result was that the Committee came to the conclusion that the conditions of nursing work are practically the same everywhere, and that if alterations were to be made it would involve the closing of some of the wards, which would be regarded by East London, and by the nurses themselves, as a grievous calamity. The Chairman said that the present attack had been made by four or five persons who cannot pretend to other than elementary knowledge of the subject in hand, whereas they have been answered by the entire medical staff, the Committee of the hospital, the nursing staff, and by Miss Florence Nightingale. The great improvements in the nursing of the London during the last ten years must be a guarantee of what will be done for their nurses in the future. Mrs. Hunter, representing the attacking party, made a long speech, which was listened to with obvious impatience, and was indistinctly heard. She shewed much courage, but had no case, and so failed on the merits. The facts were against her on all points. Mr. J. H. Buxton proposed, and Mr. Cobb, M.P., seconded, that the report of the Committee and of the sub-committee be adopted. Mr. Yatman moved, and Mr. Hunter seconded, an amendment, that in adopting the report the governors expressed a hope that the Committee would inquire into the nursing system. Mr. Yatman only secured four votes, *i.e.*, Mr. and Mrs. Hunter, Mr. Yatman, and one other governor, and his amendment was greeted with loud cries of "Not necessary!" Mr. Buxton's motion was then put and carried unanimously and with great enthusiasm. We propose to give full particulars of the report of the sub-committee next week, in the meanwhile we content ourselves with congratulating the London Hospital, its Governors, House Committee, and able staff of officers on this signal and well deserved annihilation of a faction as ill-informed as it has proved itself to be vindictive. Sir Edmund Currie summed up the whole controversy by declaring that it was "an attempt on the part of one woman —not Mrs. Hunter, by-the-bye—to knife another woman."

